# Consumption Trends During the COVID-19 Crisis: How Awe, Coping, and Social Norms Drive Utilitarian Purchases

**DOI:** 10.3389/fpsyg.2020.588580

**Published:** 2020-10-19

**Authors:** Yikai Yang, Ou Li, Xixian Peng, Lei Wang

**Affiliations:** ^1^School of Management, Zhejiang University, Hangzhou, China; ^2^Neuromanagement Lab, Zhejiang University, Hangzhou, China

**Keywords:** the COVID-19 crisis, utilitarian products, awe, coping, social norms

## Abstract

As the coronavirus disease 2019 (COVID-19) crisis continues to worsen globally, there exists a widespread enthusiasm for buying utilitarian products in the retail market, irrespective of culture or nationality. However, the questions of whether and why being involved in a public health emergency like the COVID-19 crisis could modify consumer behaviors have been underexplored by previous literature. Drawing on the theory of awe that highlights the important role in influencing human behaviors when they are facing unexpected events that transcends the frame of existing references, the present research aims to clarify the relationship between COVID-19 involvement and consumer preference for utilitarian versus hedonic products. We collected data from 512 Chinese participants (319 women; average age 29.11 years; SD = 11.89) during the outbreak of COVID-19. The results of structural equation modeling showed that COVID-19 involvement was positively related to the preference for utilitarian products (vs. hedonic products). More importantly, this relationship could be explained *via* the mediated effects of awe, problem-focused coping, and social norm compliance. The present research emphasizes the non-negligible role of public health emergencies in modifying consumer behavior and the role of awe in explaining the psychological influence of public health emergencies.

## Introduction

The outbreak of coronavirus disease 2019 (COVID-19) has been regarded as a major public health emergency, which has infected more than 10 million people and claimed over 600,000 lives worldwide as of July 20, 2020 (Johns Hopkins University, 2020). Not surprisingly, it has caused intense discussions among researchers worldwide. One key research focus is the psychological and behavioral impact of the COVID-19 crisis. For instance, it could influence human mental well-being (Ozamiz-Etxebarria et al., 2020), psychological interventions (Duan and Zhu, 2020), public emotional responses (Qian and Li, 2020), and health risk behaviors (López-Bueno et al., 2020). However, there is still limited exploration of its impact on consumer behaviors. Observably, this globally spreading pandemic and its associated strict lockdown rules are changing people’s social activities as well as their purchasing behaviors, irrespective of culture or nationality. For instance, there seems to be widespread enthusiasm for buying utilitarian products that consumers have been emptying store shelves of products such as household staples, instant food, and even toilet paper, as evidenced by the news outlets in various countries including China (Mahtani, 2020), Singapore (Altstedter and Hong, 2020), Italy (Askew, 2020), the United Kingdom (Zeltmann and Borland, 2020), and elsewhere. A critical question inspired by this phenomenon is why being involved in a public health emergency, like the COVID-19 pandemic, can spark consumers’ preferences for utilitarian rather than hedonic products.

To address this research question, we draw on the theory of awe (Keltner and Haidt, 2003) to examine how involvement in the COVID-19 crisis influences individuals’ product preferences *via* awe, problem-focused coping, and social norms. Awe is an emotional state induced by a passive, receptive mode of attention in the presence of unexpected stimuli. Awe-eliciting stimuli are often characterized by two features: perceived vastness and need for accommodation (Keltner and Haidt, 2003). As such, prior studies have consistently found that risk events with perceived vastness like tornadoes, earthquakes, and floods can trigger feeling of awe, subsequently accompanying with state need for cognitive accommodation and behavioral schema change (Keltner and Haidt, 2003; Piff et al., 2015; Anderson et al., 2018). Accordingly, the current paper proposes that experiencing the unexpected and global spreading COVID-19 may cause feeling of awe and modify consumers’ behavioral schema by facilitating more utilitarian product purchases. Moreover, we extend the theory of awe by showing that problem-focusing coping and social norms modulate the schema change procedure. As such, our focus on consumer behavior adds a much-needed perspective on the influence of public health emergencies like COVID-19 that is missing from the existing literature. The investigation on the underlying mechanism also contributes to previous literature on awe by highlighting the roles of problem-focused coping and social norms in cognitive accommodation procedure after experiencing awe.

### COVID-19 and Product Preference

According to the news outlets in various countries (Altstedter and Hong, 2020; Askew, 2020; Mahtani, 2020; Zeltmann and Borland, 2020), the outbreak of COVID-19 seems to induce more utilitarian purchases. Utilitarian products are usually employed as the means to achieve tangible outcomes (Batra and Ahtola, 1991), and thus consumers purchase them for instrumental purposes (Strahilevitz and Myers, 1998). In contrast, hedonic products are often viewed as the tools to regulate consumers’ emotions and thus consumers purchase them for entertainment or due to personal interest (Wang and Li, 2019; Wang et al., 2019). Compared to hedonic products, utilitarian products are oriented to problem-solving. As a result, purchasing utilitarian products is viewed as relatively necessary when people are involved with situations eliciting problem-solving mindsets. Previous studies have consistently shown that consumers tend to seek problem solution rather than emotion recovery when involved in the outbreak of public emergency events (Yeung and Fung, 2007; Cai et al., 2020). As the COVID-19 crisis is a typical public emergency event, being involved in it would activate people’s utilitarian mindsets of searching for ways to solve current problems. During shopping, people would tend to allocate more attention to and buy more utilitarian products, as they are more fit with people’s utilitarian mindsets induced by the COVID-19 crisis. Accordingly, we propose the main hypothesis that greater involvement in COVID-19 can elicit higher preference for utilitarian products (vs. hedonic products).

*H1*: Involvement in COVID-19 is positively related to preference for utilitarian (vs. hedonic) products.

### COVID-19 and Awe

Awe is an emotional response to the perception of a vast stimulus that transcends the frame of existing references (Keltner and Haidt, 2003; Shiota et al., 2007; Piff et al., 2015). It is a complex emotion that entails both negatively valenced feelings of threat, confusion, or anxiety, and positively valenced feelings of beauty or virtue (Keltner and Haidt, 2003; Shiota et al., 2007; Valdesolo and Graham, 2014). Increasing research has demonstrated the important role of awe in explaining various cognitive and behavioral responses, such as self-awareness (Shiota et al., 2007), time perception (Rudd et al., 2012), prosocial behavior (Piff et al., 2015), and collective action engagement (Bai et al., 2017). More relevant to this study, awe is also considered to affect consumer behaviors like advertising evaluation (Berger and Milkman, 2012) and food choice (Cao et al., 2020).

Awe-eliciting stimuli are usually characterized by two features: perceived vastness and need for accommodation (Keltner and Haidt, 2003). “Perceived vastness” specifies that the objects of awe should be vast and transcend the routine cognitive reference system. Given the global impact and terrible consequence of COVID-19, it should be considered as a risk event with perceived vastness. “Need for accommodation” means the need for cognitive accommodation and behavioral schema change to adapt to awe, which is also observable in the COVID-19 crisis. For instance, the policy of keeping social distance alters social activity schema, the increasing number of the infected and dead causes psychological burden, and the lockdown rules change working and studying patterns (Van Bavel et al., 2020). Overall, the COVID-19 crisis is qualified with the features of perceived vastness and need for accommodation to introduce feeling of awe. Therefore, with more involvement in COVID-19, people may develop greater feeling of awe.

*H2*: Involvement in COVID-19 is positively related to awe.

### COVID-19 and Problem-Focused Coping

Coping refers to the cognitive and behavioral efforts that individuals exert to manage stressful events (Folkman et al., 1986). Lazarus and Folkman (1984) distinguished two general forms of coping: problem-focused coping and emotion-focused coping. Problem-focused coping involves direct actions to resolve the stressful event or alter the source of the problem, whereas emotion-focused coping aims to manage the negative emotions associated with the stressful event (Lazarus and Folkman, 1984). Although both would be activated to manage stressful events, problem-focused coping is usually believed to play a central role in achieving effective coping results (Lazarus, 2000). By analyzing 100 assessments of coping, Skinner et al. (2003) found that problem-focused coping statements were included in almost every coping assessment. The dominance of problem-focused coping is particularly pronounced for dealing with public health emergency events. For example, problem-focused coping strategies, such as active coping (e.g., wearing face masks) and instrumental social support (e.g., getting advice from others), are more likely to be adopted in the face of SARS (Yeung and Fung, 2007). Therefore, it can be inferred that people will also form a tendency to adopt problem-focused coping when involved in the COVID-19 crisis.

*H3*: Involvement in COVID-19 is positively related to problem-focused coping.

In addition to the COVID-19 involvement itself, the awe induced by the crisis may also drive people to adopt problem-focused coping. Based on the awe theory, there exists the need for cognitive accommodation to make schema change when experiencing awe (Keltner and Haidt, 2003). Differentiated from emotion-focused coping, problem-focused coping emphasizes the cognitive effort to handle the difficult situation (Lazarus and Folkman, 1984), which is more consistent with the need for accommodation to search for effective ways to cope with stressful situations. Thus, problem-focused coping should be expected as a dominant coping means to deal with the feeling of awe caused by COVID-19. Several empirical studies have verified the link between awe and problem-focused coping. For example, feeling of awe can make people focus more on solving current problems, and as a result, people would feel that time is slowing down (Rudd et al., 2012). The core compositions of awe (e.g., threat and anxiety) could evoke a tendency to use problem-focused coping (Folkman and Lazarus, 1985, 1990). Therefore, we posit that awe can activate problem-focused coping. Given the positive link between involvement in COVID-19 and awe, we also posit that awe mediates the positive effect of involvement in COVID-19 on problem-focusing coping.

*H4*: (a) Awe is positively related to problem-focused coping; (b) awe mediates the positive effect of involvement in COVID-19 on problem-focused coping.

Coping strategies are closely related to consumption behaviors (Yi and Baumgartner, 2004). Specifically, problem-focused coping is commonly consistent with the purpose of purchasing utilitarian (vs. hedonic) products (Voss et al., 2003). Utilitarian products are usually consumed for instrumental purposes, while hedonic products are regarded as means to regulate emotion (Strahilevitz and Myers, 1998). When participants were asked to generate adjectives for utilitarian products, they tended to describe utilitarian products as problem-solving (Voss et al., 2003). Xu and Jin (2020) also demonstrated that people were more likely to perceive utilitarian products as tools to solve problems than hedonic products. Therefore, acquiring utilitarian products to solve problems is congruent with the mindset of problem-focused coping. In line with this, we propose that greater tendency to adopt problem-focused coping is positively related to preference for utilitarian products (vs. hedonic products). Combining the above discussion of the relationships among involvement in COVID-19, awe, and problem-focused coping, we also hypothesize the indirect effect of involvement in COVID-19 on product preference *via* awe and problem-focused coping.

*H5*: (a) Problem-focused coping is positively related to preference for utilitarian (vs. hedonic) products; (b) awe and problem-focused coping mediate the positive effect of involvement in COVID-19 on product preference.

### COVID-19 and Social Norms

Social norms, regarded as individual perceptions of particular group behavior as well as collective representations of acceptable group behavior (Lapinski and Rimal, 2005), specify what most people are doing or ought to do (Cialdini et al., 1990). In our research context, social norms mean behaviors adopted by most people during the COVID-19 crisis. For example, when people find that others around their belonged communities are taking efforts to prevent COVID-19, they may also choose the same protection actions. Social norms provide guidance for helping individuals to deal with uncertain or even dangerous situations (Pillutla and Chen, 1999). When exposed to risk events like SARS, people are more inclined to follow others’ plans and behaviors of others (Syed et al., 2003; Xie et al., 2011). The current infectious disease of COVID-19 is also a risk event with even more serious damages to individuals and societies. With higher involvement in such an uncertain crisis, it can be inferred that people are more likely to obey social norms.

*H6*: Involvement in COVID-19 is positively related to social norm compliance.

Besides involvement in COVID-19, the epidemic-induced awe may also promote people to obey social norms. The theory of awe suggests that awe could reduce one’s focus on the self but shift to express the self in terms of group identification (Keltner and Haidt, 2003). When individuals experience awe, they tend to perceive themselves as small parts of a large whole community. In other words, they generate a feeling of self-diminishment that individuals are powerless without society (Van Cappellen and Saroglou, 2012; Piff et al., 2015). As a result, the experience of awe leads people to be more willing to integrate into society and follow collective behaviors. Complying with social norms is an important sign that reflects the tendency to pursue group identification. Therefore, individuals who experience an induced sense of awe may be more likely to rely on social norms to guide their behaviors. Moreover, given the recognition that COVID-19 is awe-elicited, we also propose that awe mediates the direct effect of involvement in COVID-19 on social norm compliance.

*H7*: (a) Awe is positively related to social norm compliance; (b) awe mediates the positive effect of involvement in COVID-19 on social norm compliance.

Social norms have consistently been shown to be closely related to consumer behaviors. With higher willingness to comply with social norms, consumers are more likely to develop consumption behaviors that aim to pursue certain instrumental purposes, such as buying utilitarian foods for a healthy life (Mollen et al., 2013) and using sunscreen for sun protection (Mahler et al., 2008), and are also less likely to purchase products for emotional regulation purposes like reducing alcohol and tobacco consumption (Arbour-Nicitopoulos et al., 2010). With the influence of the COVID-19 crisis, protecting ourselves and our families should be the main purpose, which is a social rule recognized by most community members. Social norms drive individuals to follow this commonly recognized social rule, which is represented by purchasing utilitarian products (e.g., masks, hand sanitizers, and other necessities) to protect themselves and support their families’ daily basic needs. Therefore, we infer that social norms can encourage people to buy utilitarian products (vs. hedonic products). Combining the above discussion of the relationships among involvement in COVID-19, awe, and social norms, we also hypothesize the indirect effect of involvement in COVID-19 on product preference *via* awe and social norm compliance.

*H8*: (a) Social norm compliance is positively related to preference for utilitarian (vs. hedonic) products; (b) awe and social norm compliance mediate the positive effect of involvement in COVID-19 on product preference.

## Methodology

To test the proposed hypotheses, we conducted a cross-sectional online survey by recruiting Chinese consumers as the participants. During the survey, the participants needed to answer a series of questions regarding their perceptions of involvement in COVID-19, awe, problem-focusing coping adoption, social norm compliance, and product preference, as well as their demographic information.

### Participants

We collected 581 responses in mainland China from April 17–25, 2020, by using an online survey platform that provides functions equivalent to Qualtrics. Since this research focused on general consumers, participants with strict confinement (i.e., those who reported a history of COVID-19 or had close contact with infected patients; *n* = 6 and who were frontline workers such as healthcare workers, police officers, community workers, and volunteers; *n* = 41) were excluded in the analysis. Besides, participants (*n* = 22) with completion time deviating more than three standard deviations from the mean were also excluded. Finally, a sample of 512 individuals was obtained. Among them, 319 were women (62.30%). The average age was 29.11 (SD = 11.89) with a range from 16 to 65 years. The responses covered 29 (out of 34) provinces in China, which was relatively comprehensive. All the participants’ places of residence have reported confirmed COVID-19 infections, supporting that they were involved in the pandemic to some degree. This study was approved by the research ethics board, and informed consent was obtained from all participants.

### Measurements

The measurements consist of the following variables: COVID-19 involvement, awe, problem-focused coping, social norm compliance, product preference, and several control variables (the details are shown in Appendix A). The wording of questions, mean, and standard deviation for key variables are given in Table 1. The variables except for COVID-19 involvement^1^ were measured by the 7-point Likert-type scale.

**Table 1:** The questionnaire items, means, standard deviations, and standardized factor loadings.

Variables and items	Mean	SD	Loadings
**COVID-19 involvement (1 = totally disagree, 100 = totally agree)**			
1. I actively follow the progress of COVID-19.	79.88	24.41	0.92
2. I often browse for information on COVID-19 in the news, media, and on the internet.	80.50	23.69	0.93
3. I often talk about COVID-19 with my family and friends.	75.22	25.82	0.80
4. COVID-19 is closely related to my current life.	66.39	27.55	0.74
**Awe (1 = totally disagree, 7 = totally agree)**			
1. I sensed things momentarily slow down.	4.22	1.96	0.62
2. I felt that my sense of self was diminished.	3.37	1.90	0.67
3. I had the sense of being connected to everything.	4.17	1.91	0.69
4. I felt that I was in the presence of something grand.	4.21	1.90	0.77
5. I felt my jaw drop.	4.01	1.95	0.78
6. I felt challenged to mentally process what I was experiencing.	4.39	1.95	0.69
**Problem-focused coping (1 = totally disagree, 7 = totally agree)**			
1. I am taking steps to eliminate the problem induced by COVID-19.	5.79	1.50	0.85
2. I am actively thinking about dealing with the problem induced by COVID-19.	5.66	1.54	0.88
3. I am focusing only on the problem induced by COVID-19.	4.51	1.84	0.71
4. I am waiting for the right moment to act.	4.66	1.80	0.72
5. I am seeking advice from others.	4.96	1.70	0.70
**Social norm compliance (1 = totally disagree, 7 = totally agree)**			
1. If more people followed society’s rules, the world would be a better place.	6.36	1.20	0.87
2. People need to follow life’s unwritten rules every bit as strictly as they follow the written rules.	6.01	1.49	0.76
3. People who do what society expects of them lead happier lives.	6.13	1.38	0.87
4. Our society is built on unwritten rules that members need to follow.	6.18	1.31	0.92
5. I am at ease only when everyone around me is adhering to society’s norms.	5.87	1.54	0.75
6. I always do my best to follow society’s rules.	6.25	1.25	0.86
**Product preference (1 = totally disagree, 7 = totally agree)**			
1–5. I prefer to buy some effective/helpful/functional/necessary/practical products.	5.95	1.45	-
6–10. I prefer to buy some fun/exciting/delightful/thrilling/enjoyable products.	3.69	1.91	-

#### COVID-19 Involvement

Following the work of Qin et al. (2011), a four-item scale was developed to measure COVID-19 involvement.

#### Awe

Yaden et al. (2019) developed the Awe Experience Scale (AWE-S) to measure feeling of awe. This scale includes six subscales, each of which consists of five items. Following the suggestion by Svebak et al. (2004), we simplified the scale by choosing the item with the highest factor loading from each subscale (factor loadings ≥0.74) to compose a simplified six-item measurement scale for this study.

#### Problem-Focused Coping

Based on the Coping Orientation to Problems Experienced inventory (COPE inventory; Carver, 1997), Litman (2006) developed a brief five-item scale to measure the tendency to adopt problem-focused coping. Each item in this scale represents one dimension of the COPE inventory. We adopted this method in this study.

#### Social Norm Compliance

We adapted the Social-Norm Espousal Scale (SNES) by Bizer et al. (2014) to measure the extent to which people are willing to obey social norms. The original scale consists of 14 items. Following the suggestion by Svebak et al. (2004), we simplified the scale by choosing the six items with the highest factor loading from the original scale.

#### Product Preference

We used the Hedonic/utilitarian (HED/UT) scale by Voss et al. (2003) to capture the extent to which participants prefer utilitarian products over hedonic products. As a classic scale in consumer psychology area, it has been consistently validated by previous studies (Ogertschnig and van der Heijden, 2004; Gursoy et al., 2006; Deng et al., 2010). This scale includes 10 semantic differential response items, with five each referring to the hedonic and utilitarian dimensions of consumer attitudes. We adopted the scale and asked participants to indicate to what extent they preferred to buy products described by the 10 different semantic product features. The preference index was calculated by the mean score of preference for utilitarian products minus the mean score of preference for hedonic products. The higher the index score, the more participant preferred utilitarian products (vs. hedonic products).

#### Control Variables

To exclude the potential confounding effects, we included several control variables as covariates in the model. To control the influence of regional economic differences in China on consumer preference, we included the gross domestic products (GDPs) of participants’ residence city in 2019^2^ as the covariate. In addition, since participants might have different perceptions of the risk brought by COVID-19, we also measured their average risk preferences (Hsee and Weber, 1999). Regional governments have played an essential role in developing and executing the relevant policies and rules to control the spread of COVID-19, so we examined the influence of participants’ personal feelings of regional governments by including perceived trust in government as a control variable (Wachinger et al., 2012). Last, participants’ demographic information (e.g., education level, monthly income, and utilitarian/hedonic consumption preferences in daily life, etc.) was also included as covariates. During data analysis, we found that including these covariates did not change the results of our model in any substantial way.

#### Data Analysis

We used structural equation modeling (SEM) in Mplus version 7.3 (Muthén & Muthén; Los Angeles, CA) to test the hypothesized paths of the model. The analysis was performed in two steps. First, the measurement model was validated by first-order confirmatory factor analysis (CFA) to establish the discriminant validity of the study variables. Next, the maximum likelihood estimation was used to test the structural model. Specifically, following the Preacher and Hayes’s (2008) recommendations, the bias-corrected bootstrapping method based on 5,000 bootstraps and 95% confidence intervals were used to estimate the regression paths of the structural model and the indirect effects simultaneously. The goodness of fit of models is evaluated by the following indices (Kline, 2005): the ratio of the chi-square statistic to the degrees of freedom (*χ*^2^/*df*, acceptable if ≤3), comparative fit index (CFI, acceptable if ≥0.90), Tucker-Lewis index (TLI, acceptable if ≥0.90), root mean square error of approximation (RMSEA, acceptable if ≤0.08), and standardized root mean square residual (SRMR, acceptable if ≤0.08).

## Results

### Measurement Testing

CFA was used to examine the discriminant validity of the five key latent variables. The results showed that the hypothesized five-factor model fitted the data better (*χ*^2^/df = 3.19, RMSEA = 0.06, CFI = 0.93, TLI = 0.92, SRMR = 0.05) than the alternative models (i.e., four-, three-, two-, and one-factor models). The standardized factor loadings for all the items were statistically significant (*p*s < 0.001), ranging from 0.62 to 0.93. The absolute values of the correlation coefficients of the key variables were all less than 0.6, as shown in Table 2. Overall, the discriminant validity of our measurements was established.

**Table 2:** Correlations among the key variables.

Variables	Cronbach’s *α*	CR	1	2	3	4	5
1. COVID-19 involvement	0.87	0.91	**0.85**				
2. Awe	0.84	0.86	0.30^***^	**0.71**			
3. Problem-focused coping	0.87	0.88	0.43^***^	0.44^***^	**0.77**		
4. Social norm compliance	0.93	0.94	0.35^***^	0.26^***^	0.59^***^	**0.84**	
5. Product preference	0.93	0.93	0.25^***^	0.12^**^	0.35^***^	0.41^***^	**0.85**

CR = composite reliability. The diagonal value (in bold) is the square root of average variance extracted (AVE). Discriminate validity is confirmed if the square root of AVE for each construct is higher than the correlation coefficients between the particular construct and any other constructs. The full correlation table including both the key and control variables is presented in Appendix A, which also indicates that there is no serious concern regarding discriminate validity. ^*^p < 0.05, ^**^p < 0.01, and ^***^p < 0.001.

### Hypothesis Test

To test our proposed hypotheses, the structural model was analyzed with COVID-19 involvement served as the independent variable, awe, problem-focused coping, and social norm compliance as the mediators, and product preference as the dependent variable. All the control variables were also included in the model. Our structural model had an adequate fit: *χ*^2^/df = 2.95, RMSEA = 0.06, CFI = 0.92, TLI = 0.91, SRMR = 0.06.

**Figure 1:**
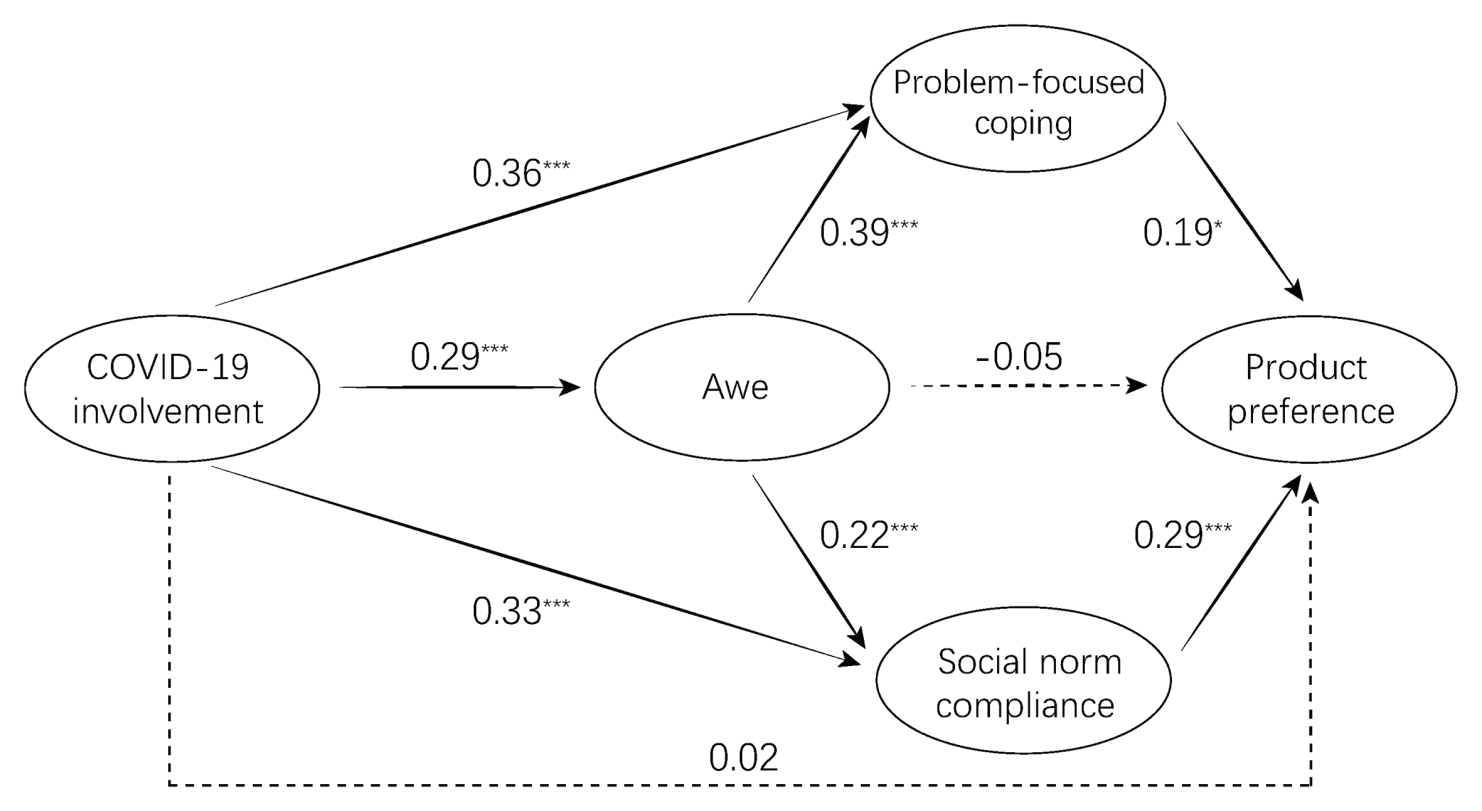
Standardized path coefficients for the structural equation model predicting product preference. Solid lines represent significant paths, and dashed lines represent nonsignificant paths. ^*^p < 0.05, ^**^p < 0.01, and ^***^p < 0.001.

The standardized path coefficients for the structural model predicting product preference are shown in Figure 1. The results showed that except for the direct relationship between COVID-19 involvement and product preference and the one between awe and product preference, all hypothesized relationships in the model were supported by the data. Though the direct effect of COVID-19 involvement on product preference was not significant, the total effect was significant with an estimate (path coefficient) of 0.20 and 95% CI [0.10, 0.31], supporting H1. As expected, COVID-19 involvement was positively related to awe (*β* = 0.29, *p* < 0.001), problem-focused coping adoption (*β* = 0.36, *p* < 0.001), and social norm compliance (*β* = 0.33, *p* < 0.001). These findings supported H2, H3, and H6. Awe was positively related to problem-focused coping adoption (*β* = 0.39, *p* < 0.001) and social norm compliance (*β* = 0.22, *p* < 0.001), which were consistent with H4a and H7a. Finally, both problem-focused coping adoption (*β* = 0.19, *p* = 0.019) and social norm compliance (*β* = 0.29, *p* < 0.001) positively associated with product preference, and thus, H5a and H8a were supported.

Table 3 also shows the results of mediation analyses. As expected, awe mediates the effects of COVID-19 involvement on problem-focused coping and on social norm compliance (i.e., indirect effects 2 and 3, respectively), which were consistent with H4b and H7b. Furthermore, both awe and problem-focused coping, and awe and social norms, also worked as successive mediators for the relationship between COVID-19 involvement and product preference (i.e., indirect effects 4 and 5, respectively), which supported H5b and H8b.

**Table 3:** Standardized parameter estimates for the direct and indirect effects for the hypothesized model of COVID-19 involvement and product preference.

Pathways	Standardized estimate	SE	95% CI	
			Lower	Upper
Direct effect: Involvement – Product preference	0.02	0.06	−0.11	0.14
Indirect effect 1: Involvement – Awe – Product preference	−0.02	0.02	−0.05	0.20
Indirect effect 2: Involvement – Awe – Problem-focused coping	0.11	0.03	0.07	0.13
Indirect effect 3: Involvement – Awe – Social norm compliance	0.06	0.02	0.03	0.16
Indirect effect 4: Involvement – Awe – Problem-focused coping – Product preference	0.02	0.01	0.01	0.05
Indirect effect 5: Involvement – Awe – Social norm compliance – Product preference	0.02	0.01	0.01	0.04

Involvement = COVID-19 involvement.

## Discussion

### Findings

Based on the theory of awe, the current paper proposes a research model to investigate the relationship between the COVID-19 crisis and a typical dimension of consumer behavior – utilitarian (vs. hedonic) product preference – as well as the mediation role of awe. We conducted an online survey with 512 participants to examine our research model and related hypotheses. The results suggest that COVID-19 involvement makes people more likely to purchase utilitarian products. More importantly, the additional test reveals that awe, problem-focused coping, and social norms explain the positive relationship between COVID-19 involvement and consumer behavior. Specifically, we find that the COVID-19 crisis, as a typical public health emergency event, is characterized by the two features of perceived vastness and need for accommodation to elicit feeling of awe. After experiencing awe, people would change their behavior schema (herein, more utilitarian purchasing) *via* cognitive accommodations from two perspectives. From the individual perspective, people could adopt more problem-focused coping strategies to address the stressful situations caused by the COVID-19 crisis. From the social perspective, feeling of awe elicited by the COVID-19 crisis could render people to focus more on social norms and comply with the rules widely recognized by society.

### Theoretical Contributions and Managerial Implications

Our research has several theoretical contributions. First, our research contributes to the literature on the psychological and behavioral influence of COVID-19. Specifically, we identify an insightful impact of the COVID-19 crisis from a consumer behavioral perspective that COVID-19 involvement can significantly increase people’s utilitarian (vs. hedonic) purchasing. Besides this direct effect, we further identify the internal mechanism. We find that being involved in COVID-19 evokes feeling of awe and subsequently enhances problem-focused coping adoption and propels compliance to social norms, which have a carrying-over effect on people’s preference for utilitarian versus hedonic products. These findings are also consistent with the emotion-cognition-behavior framework under public health emergency literature (Yeung and Fung, 2007; Xie et al., 2011) that public health emergency events like COVID-19 alter various consumer behaviors *via* affecting their emotions and triggering cognitive accommodations. For example, Sneath et al. (2009) found that disaster victims would engage in impulsive and compulsive purchasing behaviors to restore their sense of self. The U.S. Bureau of Economic Analysis recently declared that the widespread of COVID-19 crisis paralyzed consumer spending habits (Fitzgerald, 2020). Our current focus on one important type of consumer behavior – utilitarian (vs. hedonic) purchasing – supplements the extant literature on epidemic consumption behavior. However, this research stream is underexplored. Therefore, the influence on other types of consumer behavior as well as the underlying mechanisms should be an interesting aspect for future research.

Second, this study contributes to the literature on awe by strengthening the understanding of both the antecedent and outcome of awe. Differentiated from much of previous literature that has identified natural phenomena (e.g., tornadoes, earthquakes, and floods) as awe-eliciting events (Keltner and Haidt, 2003; Piff et al., 2015; Anderson et al., 2018), this research verifies that public health emergency events like COVID-19 would also evokes feeling of awe. In terms of awe’s outcome, we examine the concrete cognitive accommodation procedure by highlighting the mediated role of coping and social norms, which has beyond much of previous literature that has predominantly examined the direct cognitive and behavioral outcomes (Shiota et al., 2007; Berger and Milkman, 2012; Rudd et al., 2012; Piff et al., 2015; Bai et al., 2017; Cao et al., 2020). Specifically, we suggest that people tend to adopt more problem-focused coping to concentrate on how to solve the current problems caused by awe-eliciting stimuli, and subsequently, they would consider changing their behavioral schema (e.g., preferring utilitarian products more) to fit their mindsets of coping with the problems. Besides the individual perspective, cognitive accommodations after experiencing awe would also be executed *via* the social route. Experiencing awe can result in a diminishment of the self but shift ones’ attention to representing the self in terms of a group (Keltner and Haidt, 2003; Van Cappellen and Saroglou, 2012; Piff et al., 2015). Therefore, complying with social norms would enhance people’s behavior schema change if they find others in their communities are changing prior purchase routines. Our empirical study confirms our propositions that the relationship between awe and the preference for utilitarian products is mediated by problem-focused coping adoption and social norm compliance.

This study also has significant managerial implications in terms of providing guidelines for policymakers and marketers. First, we find that being involved in COVID-19 could induce feeling of awe to the individual, which was positively related to problem-focused coping tendency and social norm compliance. To control the spread of pandemics like COVID-19, governments first should organize specific teams to collect various problems and concerns faced by the public and then provide practical suggestions (e.g., how to wear a mask or wash hands correctly) to them. Also, as feeling of awe also leads to social norms compliance, governments should consider the power of social influence when delivering prevention policies. For example, they should highlight the importance of collectivism and social harmony in various media. From the business perspective, the finding of increased utilitarian (vs. hedonic) product consumption caused by COVID-19 involvement also provides important information for marketers, especially for e-commerce marketers. The inventories of utilitarian products should be increased to meet consumer demand resulted from higher utilitarian needs during the COVID-19 crisis. When designing promotion messages, marketers should consider adding slogans related to problem-focused coping (e.g., wash your hands and fight the virus) and social norms (e.g., everyone’s essential hand sanitizer) to attract consumers’ attention. In summary, we hope that the improved understanding of emotion-cognition-behavior under the COVID-19 crisis will help policy-makers and marketers guide consumers more effectively.

### Limitations and Future Directions

As an exploratory study, some limitations should be taken into considerations when interpreting our findings. The first limitation of our study is related to methodological design. The cross-sectional design suffers from the problem of causal inference. However, our main objective is to examine the potential contemporary relationship between public health emergencies like COVID-19 and consumer behavior, which is missing from existing literature. We still encourage future efforts to examine the delayed or long-term effect to strengthen the causal relationship by using longitude or experimental method. Also, all the measures including product preference were self-reported. Although all the scales have consistently been validated by previous studies, we encourage future studies to collect real-world sales data from retailers or examine the actual purchase behaviors of consumers to enhance the robustness of our results.

Another limitation is related to our participants’ representativeness. Especially, we used convenience sampling instead of the random sampling method to recruit participants, causing a possibility of selection bias. Most of the recent COVID-19 studies have used the convenience sampling method to recruit the participants (Ahorsu et al., 2020; Zhang and Ma, 2020). Compared with these studies, a sample size of 512 individuals was also persuasive and convincing. Despite this, the present study, as an exploratory research, could also provide evidences to some extent. Moreover, we only recruited Chinese people as participants. Chinese people were the first ones influenced by COVID-19 and were also doing well in preventing the spread of COVID-19. The protection means by the Chinese government and ordinary people have been widely adopted by other countries with different cultural backgrounds. Purchasing more utilitarian products is also a global phenomenon, as reported by the media in various countries. As such, cultural influence should not be a big concern for our research. We still call for further investigations to repeat and extend our findings in different cultural contexts.

The last limitation is related to other confounding effects. Although we have tried to exclude possible confounding effects by including participants’ demographic information, risk perception, and economic state as control variables, there are still other factors, for instance, the degree of confinement. Recent literature has suggested that COVID-19 confinement could influence health risk behaviors (e.g., higher screen exposure and lower physical activity), which may affect consume-related behaviors subsequently (López-Bueno et al., 2020). In our study, participants were experiencing the same degree of confinement during the survey. They could go to offline shopping malls with health code and there was almost no constrain for online shopping, and as a result, the confinement should not bias our findings greatly. However, as suggested by López-Bueno et al. (2020), confinement is an essential factor in the COVID-19 crisis. Similarly, future cross-country research is encouraged to investigate the effects of confinement policies by different countries on consumer behaviors as well as the underlying mechanisms.

## Conclusion

This paper supports that being involved in a public health emergency like COVID-19 has a significantly positive relationship with the preference for utilitarian products (vs. hedonic products). More importantly, the theory of awe explains this observation, according to the finding of the mediated effects of awe, problem-focused coping, and social norm compliance. Overall, the present paper highlights the non-negligible role of public health emergencies in modifying consumer behavior and also has significant managerial implications for policymakers and marketers.

## Data Availability Statement

The datasets presented in this study can be found in online repositories. The raw data related to the present study are available at https://osf.io/uvwb6/.

## Ethics Statement

The studies involving human participants were reviewed and approved by the Ethics Committee of Zhejiang University. Written informed consent to participate in this study was provided by the participants’ legal guardian/next of kin.

## Author Contributions

YY and OL conceptualized and designed the study and were involved in the data collection, analysis, interpretation, and overall writing of the manuscript. XP and LW critically revised the manuscript and gave excellent advice. All authors contributed to the article and approved the submitted version.

### Conflict of Interest

The authors declare that the research was conducted in the absence of any commercial or financial relationships that could be construed as a potential conflict of interest.

## Footnotes

^1^In the pilot study, we found that the 7-point scale might cause a serious ceiling effect and consequently changed the score range from 1–7 to 1–100.

^2^GDPs were collected from National Bureau of Statistics of China. Average risk preferences and the perceived trust in government were coded from the Chinese Family Database (a national representative sample, *N* = 40,011).

## Supplementary Material

The Supplementary Material for this article can be found online at:

https://www.frontiersin.org/articles/10.3389/fpsyg.2020.588580/full#supplementary-material
