# Supplementary material for: Consumption Trends During the COVID-19 Crisis: How Awe, Coping, and Social Norms Drive Utilitarian Purchases
Source: Front Psychol. 2020 Oct 19;11:588580. doi: 10.3389/fpsyg.2020.588580 (PMC7604535; doi:10.3389/fpsyg.2020.588580)
Supplement: Supplementary file 1 [file Table_1.docx]

APPENDIX A

**Table 1. Correlations table including both the key and control variables.**

| ***Variables*** | ***1*** | ***2*** | ***3*** | ***4*** | ***5*** | ***6*** | ***7*** | ***8*** | ***9*** | ***10*** | ***11*** |
| --- | --- | --- | --- | --- | --- | --- | --- | --- | --- | --- | --- |
| 1. COVID-19 involvement | - |  |  |  |  |  |  |  |  |  |  |
| 2. Awe | 0.30^***^ | - |  |  |  |  |  |  |  |  |  |
| 3. Problem-focused coping | 0.43^***^ | 0.44^***^ | - |  |  |  |  |  |  |  |  |
| 4. Social norm compliance | 0.35^***^ | 0.26^***^ | 0.56^*a*^ | - |  |  |  |  |  |  |  |
| 5. Product preference | 0.25^***^ | 0.12^**^ | 0.35^***^ | 0.41^***^ | - |  |  |  |  |  |  |
| 6. GDPs of the cities to which participants belonged | 0.18^***^ | 0.04 | 0.12^**^ | 0.19^***^ | 0.36^***^ | - |  |  |  |  |  |
| 7. Average risk preferences | -0.14^**^ | -0.07 | -0.14^**^ | -0.03 | -0.16^***^ | -0.18^***^ | - |  |  |  |  |
| 8. Perceived trust in government | 0.02 | 0.04 | 0.10^*^ | 0.08 | 0.07 | -0.04 | 0.09 | - |  |  |  |
| 9. Education level | -0.02 | -0.02 | -0.02 | -0.02 | -0.02 | -0.11^*^ | 0.20^***^ | 0.16^***^ | - |  |  |
| 10. Monthly income | 0.05 | -0.02 | -0.05 | -0.11^*^ | -0.05 | -0.01 | 0.07 | 0.01 | -0.09^*^ | - |  |
| 11. Utilitarian/hedonic consumption preferences in daily life | 0.07 | 0.01 | -0.01 | -0.05 | 0.02 | -0.03 | 0.04 | 0.10^*^ | -0.06 | 0.76^***^ | - |

*Note.* Variables 1-5 are key variables, and variables 6-11 are control variables. ^*^*p* < 0.05, ^**^*p* < 0.01, ^***^*p* < 0.001.
